# A set of isomeric episomal plasmids for systematic examination of mitotic stability in Saccharomyces cerevisiae

**DOI:** 10.1002/yea.3231

**Published:** 2017-03-24

**Authors:** Ruben Hohnholz, Kim Julia Pohlmann, Tilman Achstetter

**Affiliations:** ^1^ City University of Applied Sciences BremenNeustadtswall 30D‐28199BremenGermany; ^2^ Jacobs University BremenCampus Ring 1D‐28759BremenGermany; ^3^ University of BremenBibliothekstraße 1D‐28359BremenGermany

**Keywords:** Saccharomyces cerevisiae, episomal (multicopy) plasmids, mitotic stability

## Abstract

Yeast episomal shuttle vectors (YEp type) are commonly used in fundamental research and biotechnology whenever elevated product levels are desired. Their instability, however, poses an impediment not only in industrial scale fermentation. In order to analyse instability which might be linked to plasmid structure, a series of YEp type plasmids that are identical in size has been assembled, differing only in the overall arrangement of the fragments used. The performance of the eight plasmid isoforms was studied with respect to mitotic stability. While transformation efficiency in two laboratory strains of Saccharomyces cerevisiae does not differ dramatically between the eight plasmids, the plasmids do not, however, perform equally well in terms of segregational stability. Although stable at about 90% plasmid‐bearing cells in selective medium, under non‐selective conditions, three plasmid forms performed better than the other five with an up to 5.7‐fold higher stability as compared with the least favourable isoform. In a subset of four plasmids (including stable and unstable isoforms) copy numbers were determined. Furthermore the functionality of the selection marker was characterized with respect to plasmid‐derived relative *HIS3* transcript levels. No significant differences in *HIS3* transcript levels could be observed between strains carrying any one of the four plasmids. Ruling out copy number and performance of *HIS3*, the results indicate nevertheless that plasmid architecture has an impact on mitotic segregation in yeast and that construction of an expression vector should take into account that the plasmid backbone itself might already show a more or less favourable arrangement of its segments. © 2017 The Authors. *Yeast* published by John Wiley & Sons, Ltd.

## Introduction

Saccharomyces cerevisiae is a widely employed model organism in fundamental and applied research. At the same time, yeast is also a production host for biofuels, fine chemicals and solvents, and high‐value biopharmaceuticals (drugs, hormones, vaccines). Most of the latter applications make the transfer of genetic information foreign to yeast (or the amplification of homologous genes) necessary. This is accomplished with the help of plasmids of various types. Episomal multicopy plasmids (YEp type) are often exploited with an intended gene dosis effect (Romanos *et al.*, 1992; Westfall *et al*., 2012; Paddon *et al*., 2013).

Based on sequences (*ori*) of the endogenous yeast 2 μm circle plasmid, recombinant (shuttle) plasmids of the YEp type can be assembled and introduced into the host via transformation of intact yeast cells or protoplasts in order to introduce homologous or heterologous sequences of interest. The performance of such plasmids in terms of transformation efficiency and mitotic stability is highly variable (Romanos *et al*., 1992; Christianson *et al*., 1992). Published data, however, are difficult to compare, as plasmids differ widely in arrangement of their functional elements, size, copy number, selection marker and additional sequences (i.e. genes of interest). The list of variables is further extended by a strain‐specific performance in terms of transformation efficiency and mitotic stability. Apart from hints regarding stability that are not documented in any detailed way, it seems that plasmid structure beyond its ‘physical appearance’ (size, supercoiling and chromatin‐like structures) plays a role in transformation efficiency as well as plasmid retention (Caunt *et al*., 1988). Transcription of the *STB* sequences of 2 μm derivatives have been shown to have an impact on partitioning efficiency (Murray and Cesareni, 1986); likewise, transcription of an *ARS* sequence on a minichromosome (YCp type) impairs *ARS* activity, resulting in a low transformation efficiency (Tanaka *et al.*, 1994). Such a transcriptional activity might result from an inefficient transcriptional termination (‘readthrough’) of a gene or from an expression block (i.e. consisting of a yeast promoter, the desired coding sequence, and a yeast terminator) placed next to the replication origin of the plasmid interfering with its replication and partitioning. If such an interference of adjacent sequences occurs, the question arises whether this interaction is enhanced or weakened through a changed orientation of one of the fragments, which in turn could result in a weakened segregation of a certain isomeric form. Transcription–replication interference can be deleterious when encounters of DNA and RNA polymerases occur on frequently trafficked (plasmid) sections (Helmrich *et al*., 2013).

In order to investigate these effects on transformation efficiency and plasmid stability in more detail, we have devised a strategy that allowed us to assemble a YEp‐like shuttle plasmid in various isomeric forms. The performance in terms of transformation efficiency and segregational stability in two different yeast strains of these plasmids was analysed in detail. Whereas transformation efficiencies did not vary to any greater extent, dramatic differences in segregational stability were observed. Plasmid copy numbers (PCN) varied for a subset which had been analysed, although no correlation became apparent with plasmid loss data. qPCR analysis on plasmid‐derived transcripts of the selection marker *HIS3* revealed no striking differences between members of the same subset of plasmids. To our knowledge, this is the first time that such systematic analyses of plasmid stability have been carried out.

## Materials and methods

Standard molecular biology methods were employed unless otherwise stated. All enzymes were used according to the suppliers' advice.

### Strains and cultivation protocols

For plasmid assembly and amplification, Escherichia coli DH5*α* [*F*′, *recA*1, *endA*1, *hsdR*17, (r_k_^−^, m_k_^+^) *phoA*, *supE*44, *thi*‐1, *relA*1, *λ* ^−^, Ф80*lacZΔM*15 (*lacZYA* ^−^ *argF*), U169, *deoR*; Life Technologies, Darmstadt, Germany] was used. Transformation of E. coli followed published methods (Hanahan, 1983). Standard LB medium was used for the propagation of E. coli supplemented with ampicillin at 100 μg/mL (final concentration, f.c.). Solid media contained 1.5% *w*/*v* agar.

Yeast strains were SY992 [*MATα*, *ura3Δ0*, *his3Δ1*, *leu2Δ0*, *trp1Δ63*, *ade2Δ0*, *lys2Δ0*, *ADE8* (Tomlin *et al*., 2001; Euroscarf collection, Frankfurt, Germany)] and BY4742 [*MATα*, *his3Δ1*, *leu2Δ0*, *lys2Δ0*, *ura3Δ0* (Brachmann *et al*., 1998; Euroscarf collection, Frankfurt, Germany)]. Yeast was transformed in the presence of lithium acetate (LiAc), polyethylene glycol (PEG 4000, AppliChem GmbH, Darmstadt, Germany) and single‐stranded carrier DNA (from salmon testes; Sigma‐Aldrich Chemie, Munich, Germany) employing a standard protocol (Gietz and Schiestl, 2007) which included a heat shock incubation for 40 min at 42°C. For yeast propagation, YPD (1% *w*/*v* yeast extract, 2% bacto peptone, 2% *w*/*v* glucose, supplemented with adenine and uracil, 50 μg/mL f.c. each) was used. For selection of His^+^ clones, synthetic medium with 2% *w*/*v* glucose and with appropriate supplements was used (Wickerham's Mineral Medium for Yeast, *Difco Manual*, 9th edn, 1953). Solid media contained 1.5% *w*/*v* agar. Yeast cells were incubated at 30°C, liquid cultures were propagated with agitation (180 rpm).

### DNA/RNA isolation and analyses and cDNA synthesis

For the cloning, plasmid DNA was prepared from transformed E. coli DH5*α* strains with commercial kits (Plasmid Maxi Kit, QIAGEN, Hilden, Germany). DNA content and purity were analysed by UV spectroscopy in combination with agarose gel electrophoresis. Restriction profile analysis was used to identify and characterize the plasmids. The sequence of pIFC3.11 was found to match published sequences (sequencing: LGC Genomics GmbH, Berlin, Germany).

For PCN and *HIS3* transcript analysis, transformed yeast clones (SY992 carrying any one of the plasmids pIFC3.11, 3.12, 3.13, or 3.14) were grown in SD_sup_ (SD with the appropriate supplements; prewarmed) overnight (*t*_0_) or in YPDAU (YPD supplemented with adenine and uracil; prewarmed) for 72 h as described for the plasmid loss studies. Total DNA extracts were prepared out of 6 × 10^7^ yeast cells according to Moriya *et al*. (2006) with minor alterations. Total RNA was extracted employing a kit [Roti®‐Prep RNA MINI kit (Carl Roth, Karlsruhe, Germany)] following the supplier's instructions. To increase the yield of extracted RNA an enzymatic cell lysis step was introduced (100 U Lyticase for 5 × 10^7^ cells, incubated at 30°C for 20 min). Integrity of the RNA was verified by agarose gel electrophoresis with 47.5% *v*/v formamide in the sample buffer [2 × RNA Loading Dye (New England Biolabs, NEB), Ipswich, MA, USA]. Before cDNA synthesis the RNA was treated with an additional DNase I step [DNase I (RNase free), Thermo Scientific, Waltham, MA, USA] following the producer's suggestion. cDNA was prepared with the help of the ProtoScript II First Strand cDNA Synthesis Kit (NEB) using an oligo (dT)_23_ VN primer (NEB).

### Oligonucleotides

Primers (Biomers, Ulm, Germany) for fragment amplification were for the E. coli sequence RH001 5′‐AT**GAGCTC**CGCAGGAAAGAACATGTGAG‐3′ (fwd, *Sac*I restriction site underlined) and RH002 5′‐TA**GAGCTC**TTCACCGTCATCACCGAAAC‐3′ (rev), for the yeast 2 μm sequence RH003 5′‐AT**GAGCTC**TCTAGCGCTTTACGGAAGAC‐3′ (fwd) and RH004 5′‐TA**GAGCTC**AGTGCTGAAGGAAGCATACG‐3′ (rev), and for the yeast *HIS3* RH005 5′‐AT**GAGCTC**TGGCCTCCTCTAGTACACTC‐3′ (fwd) and RH006 5′‐TA**GAGCTC**CGCCTCGTTCAGAATGACAC‐3′ (rev).

PCN was assessed by qPCR producing amplicons of similar size and GC content with KP01 5′‐CTCATCCAAAGGCGCAAATC‐3′ (fwd) and KP02 5′‐ACCATCACACACCACTGAAGAC‐3′ (rev), amplifying a 113 bp fragment of the *HIS3* ORF (YOR202W). As a single copy reference a fragment (111 bp) of the chromosomal yeast gene *ENB1* (YOL158C) was amplified employing primers KP04 5′‐ACGGGATTTCGGGTCTTATTG‐3′ (fwd) and KP05 5′‐CCATCTCATTGTGGGTGGTT‐3′ (rev).

*HIS3* transcript level analyses (qPCR) were carried out with the same primers KP01 and KP02. As a reference, KP09 5′‐TGTCACGGATAGTGGCTTTG‐3′(fwd) and KP10 5′‐TTCACGTCCCGTACATACATTC‐3′ (rev) were used, amplifying a 122 bp fragment of the chromosomal yeast gene *ALG9* (YNL219C).

### DNA amplification and cloning procedures

**Figure 1:**
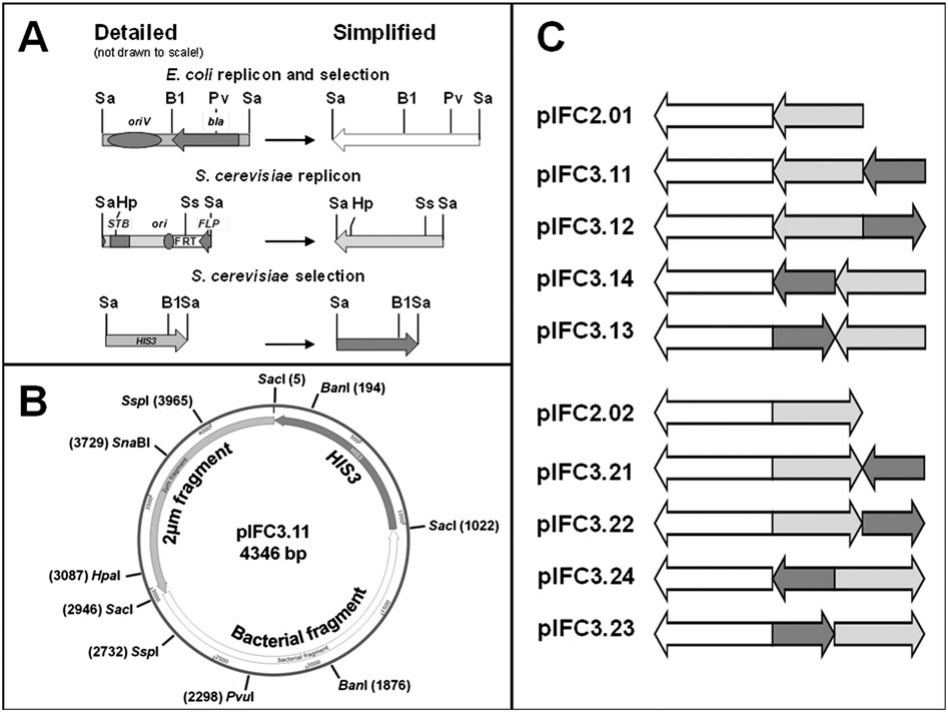
Isomeric forms of a basic E. coli‐yeast shuttle vector carrying a yeast *HIS3* marker gene. (a) (Open arrow) Bacterial fragment [1924 bp, amplified from pUG34 (GenBank: AF298784.1)] with *oriV* and *bla*, where the arrow marks the direction of transcription of the *bla* gene. (Shaded arrow) yeast 2 μm fragment [1405 bp (GenBank: J01347.1), amplified from the native 2 μm (B form) of Saccharomyces cerevisiae strain SY992 (Tomlin *et al.*, 2001)] with terminator sequences of the *REP3* gene, *STB*, *ori*, an FRT sequence, and the 3′ region of the *FLP* gene (the 3′‐end of the *FLP* coding sequences as well as its terminator), where the arrow marks the direction of transcription of the *FLP* gene. (Black arrow) S. cerevisiae *HIS3* gene (1017 bp, YOR202W, amplified from pUG34), where the arrow marks the direction of transcription of the *HIS3* gene. Sa, *Sac*I; B1, *Sna*BI; Pv, *Pvu*I; Ss, *Ssp*I; Hp, *Hpa*I. (b) pIFC3.11, simplified drawing made with Snapgene Viewer. (c) pIFC plasmid family (schematic drawing); pIFC2.01–pIFC2.02 plasmids are 3329 bp long, pIFC3.11–pIFC3.24 are 4.346 bp.

With *Taq* polymerase (Vent Polymerase, NEB) DNA was amplified in polymerase chain reactions using various templates (see Figure 1). The bacterial and the yeast 2 μm fragments were cleaved with *Sac*I, ligated (the bacterial amplicon was dephosphorylated prior to its ligation) and used to transform E. coli. *Amp* ^R^ transformants were analysed for their plasmid content using *Sac*I, *Pvu*I and *Ssp*I, yielding the plasmids pIFC2.01 and pIFC2.02 (Figure 1).

The *HIS3* fragment was PCR‐amplified, and the amplicon was cleaved with *Sac*I and agarose gel purified with the help of a commercial kit (PCR purification kit, Roche, Mannheim, Germany). Plasmid pIFC2.01 and pIFC2.02, respectively, were linearized with *Sac*I in a partial digest, dephosphorylated and gel purified with the help of a commercial kit. The purified fragment and either one of the linearized and dephosphorylated plasmids pIFC2.01 or pIFC2.02 were ligated and used to transform E. coli. *Amp* ^R^ transformants were analysed for their plasmid content using *Ban*I yielding in the pIFC3.1 and pIFC3.2 plasmid series (Figure 1).

### Estimation of PCNs

PCNs were estimated by comparing the relative quantity of plasmid derived *HIS3* with endogenous *ENB1*. Calculations were done employing the following equation:

(1) $$\mathrm{PCN}\;\mathrm{per}\;\text{haploid genome}\;=2^{\left(C_{t}\;\mathrm{ENB}1-C_{t}\;\mathrm{HIS}3\right)}$$

where C_t_ *ENB1* and C_t_ *HIS3* are the PCR cycle numbers at threshold points of their respective PCR amplification curves. qPCR was performed with the PerfeCTa® SYBR® Green FastMix (Quanta, Beverly, MA, USA). Detection system and analysis software were the Mastercycler® RealPlex^2^ and *realplex* software version 2.2 (Eppendorf, Hamburg, Germany). For each plasmid, three clones with three biological and two technical replicates were analysed**.**

### Estimation of relative transcript levels

The relative levels of *HIS3* and *bla* transcripts were calculated by comparing the relative levels of plasmid derived transcripts to the plasmid level. Normalization was done with the endogenous *ALG9*. The calculations were:

(2) $$\text{relative transcript level}=\left(C_{t}\;\mathrm{ALG}9-C_{t}\;\mathrm{HIS}3\right)$$

(3) $$\text{plasmid level}=\left(C_{t}\;\mathrm{ENB}1-C_{t}\;\mathrm{HIS}3\right)$$

where C_t_ *ALG9*, C_t_ *ENB1* and C_t_ *HIS3* are the PCR cycle numbers at threshold points of the PCR amplification curves of *ALG9* and *HIS3* polyA transcripts, and of *ENB1* and *HIS3* genes, respectively. The relative level of transcripts per plasmid was calculated by**:**

(4) ratio=relative transcript level/plasmid level

Furthermore, numbers were related to plasmid carrying His^+^ cells as determined in replica plating assays (see below).

### Plasmid loss studies

Plasmid loss studies followed closely a published protocol (Christianson *et al*., 1992). For each plasmid and each strain, three His^+^ yeast transformants were precultured overnight in SD_sup_. The following morning, cells were diluted 1:1000 in fresh SD_sup_ (*t*_0_) or 1:2000 in fresh YPDAU (*t*_0_). Every 24 h for 5 days in a row, cultures in selective medium were diluted back 1:1000 in fresh SD_sup_, whereas cultures in non‐selective medium were diluted back 1:4000 in YPDAU.

From each YPDAU and SD_sup_ (*t*_0,_ *t*_24,_ *t*_72_, *t*_120_) flask, a sample was withdrawn, diluted and plated out on solid YPDAU (in triplicates) and incubated at 30°C. After 2 days of incubation at 30°C, YPDAU plates were replica plated on solid SD_sup_ to identify plasmid‐carrying His^+^ clones.

## Results and discussion

Gene analysis and protein production in yeast frequently make use of available (multicopy) plasmids. Such plasmids serve as universal tools in the laboratory without being optimized for their performance with respect to their transformation efficiency and mitotic stability. Here we present data with the help of a systematically developed plasmid family which demonstrate that, at least for segregational stability, plasmid architecture matters. All members of the plasmid family presented have the same size of 4346 bp. They all carry the same three fragments, i.e. sequences allowing for propagation (*oriV*) and selection (*bla*) in E. coli, a segment of the yeast 2 μm with an origin of replication (2 μm *ori*) and a sequence necessary for efficient segregation (*STB*), and a copy of the yeast *HIS3* gene for selection (Figure 1A). Fragments vary in order and/or orientation, and thus, the plasmids might be considered isomers (Figure 1B and C). Potential differences in transformation efficiency and segregational stability are thus reduced to the order and orientation of the sequences described. To our knowledge, this set of plasmids allows for the first time a thorough analysis of the arrangement of functional sequences.

Each member of the set of plasmids carrying a copy of the *HIS3* gene (Figure 1) was analysed for its transforming capacity and retention in the cells over an extended period. The *HIS3* gene was chosen for selection because the complementing fragment is rather small and, at the same time, the growth rate of plasmid‐carrying cells was reported not to be affected to a larger extent (Chee and Haase, 2012; Karim *et al*., 2013). To rule out strain‐specific effects, the two common laboratory strains BY4742 and SY992 were both used as hosts. Transformation efficiencies did not differ significantly between the plasmids (0.83–1.65 × 10^6^ for SY992 and 1.0–2.2 × 10^6^ for BY4742 per μg of DNA and 1 × 10^8^ cells, not shown) using the LiAc/single‐stranded carrier DNA/PEG method (Gietz and Schiestl, 2007), which is well within the expected range of the published 1 × 10^6^ transformants per μg plasmid DNA per 10^8^ cells (Gietz and Schiestl, 2007; Mitrikeski, 2015).

Three independent transformants for each strain and each plasmid were analysed for their mitotic stability, with an average of 505 individual colonies (on 3 × 3 plates) being replica plated at each given time point (see Material and Methods). In SD_sup_, all eight plasmids showed a constant fraction of plasmid‐bearing cells of at least 85% for at least 44 generations (data not shown except for pIFC3.11–pIFC3.14 for selected time points, Table 2). It is in accordance with the literature that, even under selective conditions, not 100% of growing cells retain YEp plasmids (e.g. Ugolini *et al*., 2002).

While the mitotic stability of the eight plasmids after 24 h in rich medium (between 10 and 13 generations) was still similar (68–80%, data not shown), a 2.4‐fold difference in plasmid‐bearing cells (26–62%) was already observed after 72 h (29–39 generations). In order to mimic the situation of an industrial process, which accumulates 50–60 generations from the primary seed lot through the secondary seed lot and preculture to the final large‐scale fermentation, the experiments were extended for a total of 120 h. After these 120 h (48–65 generations), in the best of all cases (pIFC3.14), just over half of the cells had lost the plasmid, returning to the His^−^ phenotype, whereas in the worst case (pIFC3.21), <10% of the cells retained the plasmid (Table 1).

**Table 1:** Mitotic stability of plasmid isomeric forms in yeast strains SY992 and BY4742.

	SY992			BY4742		
Vector^a^	Mean value for three transformants after *g* generations^b^					
	Percentage (*P*) plasmid‐carrying cells^c^					
	After *g* generations^d^			After *g* generations^d^		
	Non‐selective		Plamid loss rate^e^	Non‐selective		Plasmid loss rate^e^
	*g*	*P*	(× 10^−2^)	*g*	*P*	(× 10^−2^)
pIFC3.11 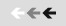	0	95.6 ± 0.7		0	87.5 ± 3.0	
	37	26.7 ± 4.9		39	27.8 ± 6.1	
	61	10.2 ± 1.8	3.6	65	14.5 ± 7.4	2.7
pIFC3.12 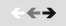	0	96.0 ± 1.5		0	92.9 ± 2.9	
	35	43.4 ± 2.8		36	45.1 ± 7.4	
	58	27.2 ± 5.3	2.0	60	25.8 ± 6.8	2.1
pIFC3.13 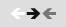	0	85.3 ± 4.3		0	88.2 ± 4.6	
	35	45.4 ± 11.2		37	62.3 ± 10.1	
	59	31.9 ± 8.9	1.7	62	43.6 ± 2.7	1.1
pIFC3.14 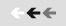	0	88.4 ± 0.3		0	89.1 ± 1.2	
	39	52.6 ± 12.0		39	58.9 ± 9.5	
	65	34.1 ± 5.1	1.5	65	46.0 ± 2.6	1.0
pIFC3.21 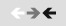	0	87.9 ± 6.4		0	90.0 ± 2.5	
	29	25.4 ± 5.1		31	26.4 ± 10.3	
	48	9.3 ± 6.4	4.6	51	4.6 ± 3.6	5.7
pIFC3.22 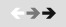	0	89.1 ± 2.1		0	91.0 ± 2.2	
	34	40.3 ± 13.0		34	46.3 ± 7.1	
	57	33.7 ± 1.7	1.7	57	37.6 ± 3.4	1.5
pIFC3.23 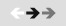	0	87.9 ± 1.9		0	91.6 ± 1.5	
	34	35.5 ± 5.2		35	47.5 ± 15.6	
	56	18.2 ± 4.7	2.8	58	24.1 ± 9.7	2.3
pIFC3.24 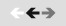	0	86.0 ± 0.2		0	86.8 ± 1.8	
	30	35.2 ± 9.3		32	41.4 ± 4.9	
	51	14.6 ± 3.9	3.4	53	14.4 ± 4.0	3.3

a Vector schemes: see Material and Methods and Figure 1.

b Three individual His^+^ transformants for each vector were inoculated into SD_sup_. OD_600_ was measured and 100 μL containing theoretically 150 cells was plated out immediately on YPDAU plates in triplicate. The colonies on the YPDAU agar were counted and then scored for plasmid retention by replica plating onto selective agar. The mean and standard deviation of the percentage of plasmid‐carrying cells in the inoculum was calculated (*P*). Standard deviation was calculated by standard methods and reflects the deviation of the three individual transformants.

c *P* was calculated by analysing clones from replica plating. On average, 505 cfu for each particular time and transformant were analysed, but at least 227 cfu. Plasmid‐carrying and plasmid‐free cells had the same doubling time in SD_sup_ and YPDAU (data not shown), which has been reported before (Christianson et al., 1992; Futcher and Cox, 1984).

d The doubling time (*d*, in hours) was calculated by diluting a stationary culture grown in SD_sup_ 1:100 into YPDAU and fresh SD_sup_, respectively. OD_600_ was measured over a period of 8 h and *d* was calculated from the data obtained using the culture's viable cell concentration (*C*1, *C*2) in exponential growth phase:

(1) $$d=\frac{\frac{1}{\ln\left(C2\right)-\ln\left(C1\right)}}{t_{2}-t_{1}}$$

and *g* = *t*(*d*)^−1^, with *t* = 0, 24, 72 and 120 h.

e Plasmid loss rate was determined after Gibson *et al*. (1990), with *I* being the initial percentage of His^+^ cells (*t*_0_) and *F* being the percentage of His^+^ cells after *N* generations:
(2) $$\text{plasmid loss rate}=1-{\left(F/I\right)}^{1/N}$$

The two plasmids pIFC3.12 and pIFC3.23 exhibit a plasmid loss rate between 2.0 and 2.8 × 10^−2^ (Table 1), corresponding to a plasmid loss of 2.0–2.8% per generation. These values are expected for YEp plasmids (Futcher and Cox, 1984; Christianson *et al*., 1992).

The isoforms pIFC3.11 and pIFC3.24 performed a little less well in segregating the plasmids stably from generation to generation with a loss rate of 2.7 to 3.6 × 10^−2^. With <10% His^+^ cells after 120 h, pIFC3.21 performed significantly worse than the other plasmids. The loss rate for pIFC3.21 resembles that of an unstable *ARS* vector (Futcher and Cox, 1984). It should be noted that the doubling time and thus the generation time after 120 h is lower in cells transformed with pIFC3.21 (and likewise pIFC3.24) than that of the other six plasmids (Table 1).

In comparison, the three plasmids pIFC3.13, pIFC3.14 and pIFC3.22 exhibit the highest stability with plasmid loss rates between 1.0 and 1.7 × 10^−2^ per generation (Table 1). Despite being isomers, there are highly significant differences in the mitotic stability between the plasmids. While the transformation was similarly efficient for all eight plasmids, the prolonged maintenance and faithful segregation to the progeny over 5 days in non‐selective medium resulted in notable differences in plasmid loss, with pIFC3.14 exhibiting the lowest and pIFC3.21 the highest instability differing by a factor of 3.0 in SY992 and even by 5.7 in BY4742. This was the case for all three individual transformants, reflected by the small standard deviation in Table 1.

The arrangement 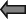 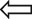 (head‐to‐tail arrangement of yeast marker and bacterial fragment, Figure 1C) is unfavourable, leading to the least stable segregation (pIFC3.11 and pIFC3.21, Table 1). Likewise, an orientation 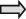 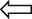 (head‐to‐head arrangement of the 2 μm fragment and bacterial fragment, Figure 1C) decreased the mitotic stability without selective pressure (pIFC3.23 and pIFC3.24, Table 1). The ideal plasmid in this study had a head‐to‐tail arrangement of the 2 μm fragment and the bacterial fragment (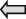 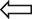) with the *HIS3* gene near the STB region (head) of the 2 μm fragment, disregarding its orientation (pIFC3.13 and pIFC3.14). Arranging the *HIS3* fragment next to the bacterial sequence in a head‐to‐head fashion 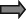 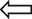 led in three out of four cases to an increased stability (e.g. pIFC3.11 vs. pIFC3.12 and pIFC3.21 vs. pIFC3.22, Table 1).

In a subset of the plasmids described in Table 1, namely pIFC3.11 through pIFC3.14, PCNs were assessed in SY992 cells grown in SD_sup_ overnight (*t*_0_) and in cells propagated in YPDAU for 72 h employing a qPCR protocol with total DNA as a template whereby *ENB1* served as single copy genomic control. After 72 h (or 35–39 generations, Table 1) only 26.7% (pIFC3.11) to 52.6% (pIFC3.14) of the cells retained a His^+^ phenotype. As His^+^ cells represent a minority in the population after prolongated, non‐selective propagation, further calculations were done considering only the plasmid‐carrying cells. qPCR analysis revealed an up to two times lower PCN in cells propagated in YPDAU in the described fashion when compared with cells grown in SD_sup_. PCNs varying between 15 and 34 copies per cell when grown in YPDAU did not reflect the respective segregational instability; neither does an initial high PCN explain the differences in plasmid loss over time (see Table 2). This neither confirms nor refutes the common notion that segregational instability seems affected by PCNs, with high copy numbers resulting in higher stability (Caunt *et al*., 1988; Futcher and Cox, 1984). On the other hand, neither does a reduced metabolic burden as a result of lower PCNs positively affect mitotic stability.pIFC3.11 and pIFC3.12 having a similar PCN per His^+^ cell, but instabilities are significantly different (Table 2).

**Table 2:** Plasmid copy numbers (PCNs) and relative *HIS3* transcript levels in His^+^ cells (strain SY992) carrying members of the plasmid subset.

Plasmid pIFC	SD_sup_ (*t*_0_)			YPDAU (*t*_72_)		
	His^+^ cells (%)	PCN^a^	*HIS3* ^a^ (± *s*)	His^+^ cells (%)	PCNs^a^	*HIS3* ^a^ (± *s*)
3.11	95.6	41	1.67 (0.12)	26.7	34	0.51 (0.02)
3.12	94.9	61	1.37 (0.07)	43.4	30	0.60 (0.04)
3.13	85.3	23	1.72 (0.07)	45.4	20	0.77 (0.10)
3.14	88.4	28	1.39 (0.16)	52.6	15	0.57 (0.11)

Cells were grown [in SD_sup_ overnight *t*_0_; in YPDAU for 72 h (corresponding to 37, 35, 35 and 39 generations, respectively) according to the plasmid loss protocol] and nucleic acids were prepared as described. PCNs and relative transcript levels were determined as described in Materials and Methods. *HIS3* transcript data shown are the mean results of three His^+^ clones with three biological and two technical replicates [± standard deviation (*s*)]. His^+^ cell data as listed in Table 1 for YPDAU. Numbers of total relative *HIS3* transcript levels were divided by PCNs for comparison (PCN twice as high is expected to generate double the amount of transcript).

a PCNs and relative *HIS3* transcript levels per plasmid in His^+^ cells, e.g. if PCN was determined to be 20, but only 33% of cells were His^+^, those cells carried indeed 60 plasmids and 67% no plasmid (i.e. had lost the plasmid).

*HIS3* transcripts, seemingly the only plasmid‐derived transcripts, relevant for selection and survival of the cells in SD_sup_, were compared. In cells grown under selective conditions, 2‐ to 3‐fold higher relative transcript levels (per plasmid) were observed when compared with cells grown in YPDAU (Table 2). In the latter condition, the cells do not depend on the selection marker, although transcription is obviously not entirely stopped. Furthermore, plasmid‐borne *HIS3* polyA transcript levels do not vary to a major extent when the four members of the subset are compared. This is true for cells grown in SD_sup_ or in YPDAU (Table 2). Taken together, a lack of function of the *HIS3* gene copy contained in the plasmids seems unlikely to be the reason for the loss observed.

Plasmid size, which is known to be a factor in instability (Futcher and Cox, 1984), and unfavourable sequences can also be excluded to be the reason for the differences in segregational stability seen between the isoforms of our plasmid. As reasoned above, differing PCNs and reduced expression of the selection system (in all isoforms analysed) cannot explain the loss. The reason thus lies in the arrangement and the orientation of the fragments, pinpointing to the junctions between the fragments as being more critical than the fragments themselves.

Pervasive transcription, known to be a factor in chromosomal gene regulation (Porrua and Libri, 2015), could be imagined for plasmids as well. From the supposedly neutral bacterial fragment and using a cryptic yeast promoter, a massive transcription has been reported which extends to neighboring yeast sequences in a S. cerevisiae plasmid (Marczynski and Jaehning, 1985). Such transcriptions might impair functions of those sequences, e.g. 2 μm *ori*, vital for plasmid maintenance in yeast. Furthermore, the replication and transcription machineries working simultaneously on the same template may collide more or less frequently if the fragments are arranged unfavourably (Prescott and Proudfoot, 2002; Helmrich *et al*., 2013).

Series of expression plasmids were developed and analysed with varying selection markers and promoters (e.g. Sikorski and Hieter, 1989; Fang *et al*., 2011; Da Silva and Srikrishnan, 2012). In those previous studies, however, arrangement of functional sequences was not taken into consideration. From our study, we were able to show for the first time that, by varying systematically the architecture of a plasmid, one can greatly influence its segregational stability in yeast, at least for the two used laboratory strains (SY992 and BY4742) and the used marker (*HIS3*) in non‐selective medium. Considering that rich medium favours growth rate (data not shown) and, at the same time, is cheaper than selective medium on the industrial scale, enhancing stability in non‐selective medium is highly desirable.

As for the reason why some isoforms get lost much faster than others under non‐selective conditions, we can only speculate. For instance, divergent nucleosome placements on the eight plasmid isoforms (as far as nucleosomes get correctly installed on plasmids at all) could affect transcription termination. Likewise, transcription–replication collisions and pervasive or cryptic transcription could interfere with the function of elements (*STB*, *ori*) on the 2 μm fragment important for segregation, which has been shown for a forced transcription on *STB* (and potentially *ori*) sequences in YEp type plasmids (Murray and Cesareni, 1986).

In conclusion, the pIFC plasmid set presented here might ultimately serve for the investigation of the stability of a plasmid carrying a heterologous or homologous gene in different positions, aiming at an optimized plasmid performance. Based on our findings, the assembly of such a plasmid should take into account that its backbone already shows a favourable or less favourable organization of its functional sequences. Even though no gene of interest has yet been introduced into our vectors, it is reasonable to assume that, in some configurations, destabilizing effects may become even more pronounced.
